# Institutional Needs

**Published:** 1914-09-12

**Authors:** 


### Institutional Needs.

### REVERSIBLE RUGS.

The Art Weavers' Guild, of 36 Wessex Works, Kidderminster, are now offering, apparently at scarcely more than cost price so as to keep their works going at any rate on part time, a special rug for the use of ambulance stations and hospitals. As the Guild is doubtless aware, rugs are not found in hospital wards, though nurses' bedrooms often contain them, and those who are furnishing convalescent homes for the wounded or equipping ambulance stations will be interested in their "Britrusance" rug, which measures 4 ft. 6 in. by 2 ft., and is reversible, soft to the tread, and guaranteed capable of hard wear. To those furnishing homes and institutions for the reception of convalescents from the hospitals the rugs may therefore be specially commended. The price is given as 4s. each, carriage paid, and a reduction is obtainable for quantities. Samples can be sent for inspection, and the colours are blended tones of blue, grey, green, rose, or red. The Guild states that it employs only British materials and British labour, and those in need of rugs and carpets for the convalescent homes now improvised in connection with the war could not do better than to test the Guild's goods for themselves.

